# Supplementary material for: Causal Relationship between Aging and Anorexia Nervosa: A White-Matter-Microstructure-Mediated Mendelian Randomization Analysis
Source: Biomedicines. 2024 Aug 16;12(8):1874. doi: 10.3390/biomedicines12081874 (PMC11351342; doi:10.3390/biomedicines12081874)
Supplement: Supplementary file 1 [file biomedicines-12-01874-s001.zip › Supplementary Figure/Figure S9/Leaveoneout plot of MR analysis for ubm-b-1639 to AN.pdf]

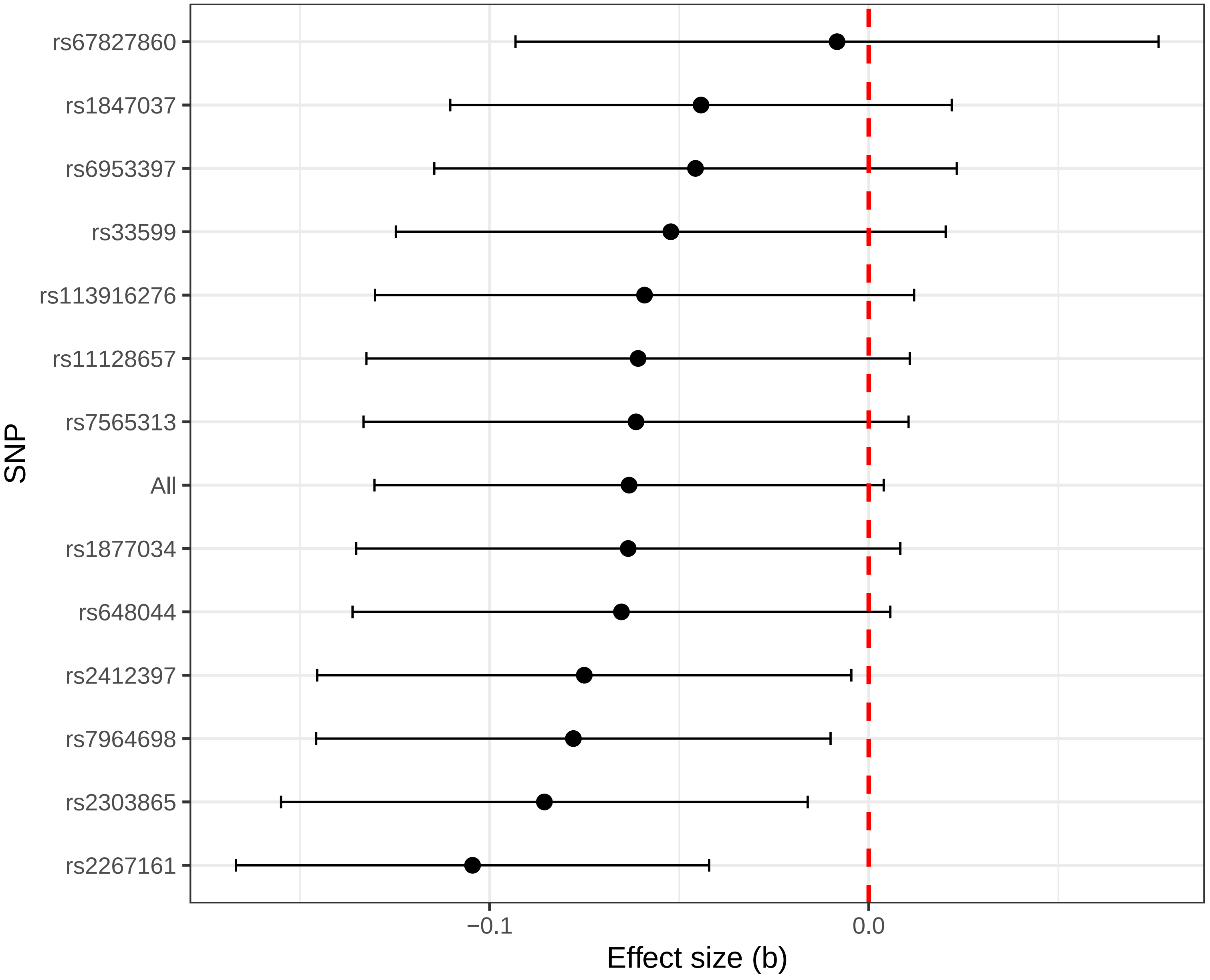

rs67827860
rs1847037
rs6953397
rs33599
rs113916276
rs11128657
rs7565313
All
rs1877034
rs648044
rs2412397
rs7964698
rs2303865
rs2267161
SNP
−0.1
0.0
Effect size (b)
